# Improving alcohol health literacy and reducing alcohol consumption: recommendations for Germany

**DOI:** 10.1186/s13722-023-00383-0

**Published:** 2023-05-09

**Authors:** Jakob Manthey, Daša Kokole, Steffi Riedel-Heller, Gill Rowlands, Ingo Schäfer, Georg Schomerus, Renate Soellner, Carolin Kilian

**Affiliations:** 1 Center for Interdisciplinary Addiction Research (ZIS), Department of Psychiatry and Psychotherapy, University Medical Center Hamburg-Eppendorf (UKE), Martinistraße 52, 20246 Hamburg, Germany; 2 Department of Psychiatry, Medical Faculty, University of Leipzig, Semmelweisstraße 10, 04103 Leipzig, Germany; 3 Department of Health Promotion, CAPHRI Care and Public Health Research Institute, Maastricht University, POB 616, 6200 MD Maastricht, The Netherlands; 4 Public Health Sciences Institute, Campus for Ageing & Vitality, Westgate Rd, Newcastle Upon Tyne, NE4 6BE UK; 5 Institute of Social Medicine, Medical Faculty, Occupational Health and Public Health (ISAP), University of Leipzig, Philipp-Rosenthal-Str. 55, 04103 Leipzig, Germany; 6 Institute for Psychology, University of Hildesheim, Universitätsplatz 1, 34414 Hildesheim, Germany; 7 Centre for Addiction and Mental Health (CAMH), Institute for Mental Health Policy Research (IMHPR), 33 Ursula Franklin Street, Toronto, ON M5S 2S1 Canada

**Keywords:** Alcohol, Health literacy, Public health policy, Alcohol control policy, Taxation, Availability, Brief intervention

## Abstract

**Background:**

Although the detrimental health effects of alcohol are well established, consumption levels are high in many high-income countries such as Germany. Improving alcohol health literacy presents an integrated approach to alcohol prevention and an important complement to alcohol policy. Our aim was to identify and prioritize measures to enhance alcohol health literacy and hence to reduce alcohol consumption, using Germany as an example.

**Methods:**

A series of recommendations for improving alcohol health literacy were derived from a review of the literature and subsequently rated by five experts. Recommendations were rated according to their likely impact on enhancing (a) alcohol health literacy and (b) reducing alcohol consumption. Inter-rater agreement was assessed using a two-way intra-class correlation coefficient (ICC).

**Results:**

Eleven recommendations were established for three areas of action: (1) education and information, (2) health care system, and (3) alcohol control policy. Education and information measures were rated high to increase alcohol health literacy but low to their impact on alcohol consumption, while this pattern was reversed for alcohol control policies. The ratings showed good agreement (ICC: 0.85–0.88).

**Conclusions:**

Improving alcohol health literacy and reducing alcohol consumption should be considered complementary and become part of a comprehensive alcohol strategy to curb the health, social, and economic burden of alcohol.

## Background

Alcohol consumption is prevalent in most middle- and high-income countries [1] and related to a wide set of health, social and economic consequences [2–4]. Several recent modelling studies have suggested that improved alcohol management in primary health care and increased alcohol taxation would have sizeable impacts on consumption and health consequences [5–7]. With few exceptions, most countries make little to no progress in reducing alcohol consumption and its consequences, contrasting international goals to lower alcohol consumption [8].

According to the World Health Organization, the availability and affordability of alcoholic beverages are key determinants for alcohol use [9]. Theoretically, lawmakers can restrict these two domains relatively easy but it needs to be acknowledged that the implementation of alcohol control policies require public and political support. A 2015 European survey showed that alcohol control policies which restrict the availability and affordability of alcoholic beverages were found to be more acceptable in countries where such policies are already implemented and among people who abstain from alcohol use [10]. Moreover, knowledge of alcohol use as a risk factor for cancer predicts support for alcohol control policies [11, 12]. Thus, in order to achieve a sustainable reduction of alcohol consumption and attributable harm, it seems prudent to change not only the physical (i.e., availability and affordability), but also the social environment (i.e., norms) as well as the individual risk perception.

Alcohol health literacy (AHL) can serve as a vehicle to consider all relevant determinants for a sustainable reduction of alcohol use. The core attributes of AHL lie in the individual and have been described as the capacity „to obtain, process, and understand knowledge about alcohol content, units, strengths, and harms” [13]. This explicitly covers the knowledge of health risks, but also the understanding of standard drinks, which is a precondition to pour alcoholic drinks according to the desired amounts. Thus, AHL is a specific category of a general health literacy concept, which is commonly defined as “the motivation, knowledge and competencies to access, understand, appraise and apply health information in order to make judgments and take decisions in everyday life” [14].

**Fig. 1:**
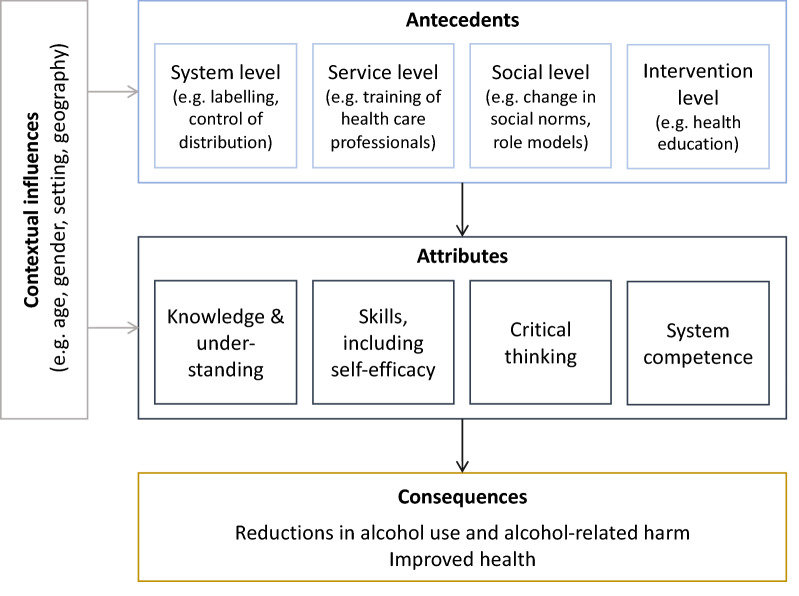
Conceptualization of Alcohol Health Literacy, adapted from [13]

According to a literature-driven theoretical conceptualization of AHL [13], the core AHL attributes as described above are embedded in social and systemic environments—called antecedents—which determine the individuals’ AHL: social norms (social environment) but also labels on alcoholic beverages, health care and educational systems (systemic environments) can work as facilitators or as barriers for establishing low-risk alcohol use patterns or abstinence. Lastly, AHL explains individuals’ decision to drink or not to drink and predicts health outcomes, which are conceptualized as AHL consequences (see Fig. 1).

Given the insufficient progress in reducing alcohol use and consequences and the relevance of AHL to achieving these aims, this contribution aimed to identify and prioritise measures to (a) increase AHL and (thereby) (b) lower alcohol consumption.

We chose Germany as a possible target for these measures given several reasons: First, per capita alcohol consumption in Germany is well above the global as well as European average [1] and the population experiences high levels of alcohol-attributable morbidity [15] and economic burden [16]. Second, Germany has not made any progress in the implementation of alcohol control policies in the past decades. While other European countries have successfully cut alcohol consumption by implementing strict alcohol control policies (see e.g., [17–19]), Germany underperforms in several alcohol policy domains, such as availability and marketing [20]. Third, the importance of public support for the sustainability of policies could be observed in the German federal state Baden-Württemberg. Despite achieving the intended effects of reducing hospitalisations among youth, a ban of nighttime sales hours was introduced in 2010 and then lifted in 2017 after a change in the government [21]. Fourth, general health literacy is considered to be low in Germany—in particular among vulnerable groups [22]—but there appears to be little empirical evidence with regards to the manifestation of AHL in the German population.

## Methods

To identify measures that improve AHL and/or lower alcohol consumption, the following three areas of action were considered: education, health care and policy. These three areas reflect the determinants for developing AHL, as described in Fig. 1. Specifically, we generalized the four antecedents and derived the three areas of action that are relevant for developing AHL and/or for reducing alcohol consumption.

For each of the three areas, the lead and last author searched for measures that have been empirically linked to either improved awareness of alcohol health risks or decision-making, i.e., changes in alcohol consumption. This involved a brief search on PubMed for systematic reviews and meta-analyses using keywords describing the measure of interest (e.g., availability). Additional studies were identified through input from the experts. In addition to empirically validated measures, we also considered the environment that constitutes a precondition to the success of other measures, for example the resources required to improve health care provision.

For a two-stage survey, we invited five experts (GR, SRH, IS, GS, RS) to (1) review the recommendations and (2) rate the recommendations with regards to the likely impact on (a) improving AHL and (b) reducing alcohol consumption. In the first stage, the experts were not only given the opportunity to refine the recommendations but also to provide additional measures that were not covered in the presented list. In the second stage, the experts were asked to rate the list of recommendations with respect to the expected impact on AHL and alcohol consumption on a scale from 1 (no impact) to 10 (very high impact). The selection of the five experts was driven by the consideration to include persons with long-standing research activities in the field of health literacy and addiction, as well as expertise in prevention and therapy. We also aimed to include both national and international expertise and a balanced gender distribution. No invited expert declined to participate in this exercise.

To evaluate the inter-rater agreement of the five experts for the final list of recommendations, a two-way intra-class correlation coefficient (ICC) was calculated with the ICC function of the ‘psych’ package [23] in R version 4.1.2 [24]. The measure of agreement indicates the proportion of variance in the ratings explained by the variation across raters. We do not assume a generalization of the sampled experts to a larger population of experts, which is why the raters were treated as fixed effect according to the recommendations by Shrout and Fleiss [25].

## Results

**Fig. 2:**
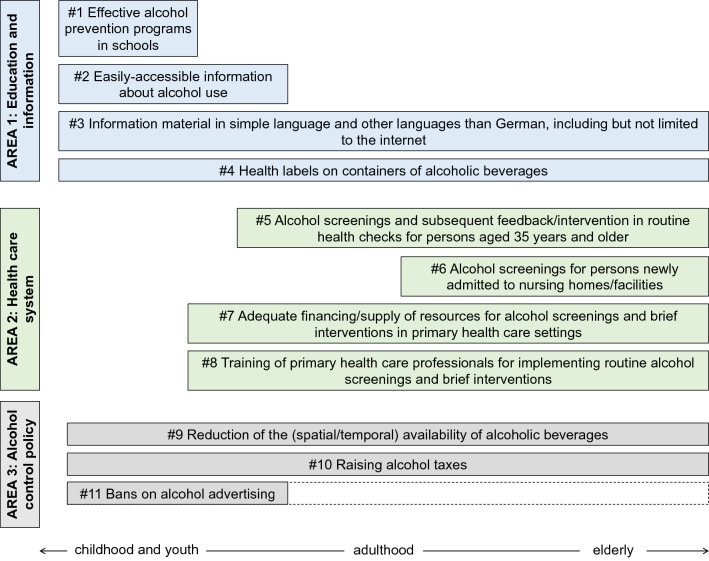
Overview of the eleven recommendations to strengthen alcohol health literacy and to reduce alcohol consumption, grouped by their area of action. The recommendations partly address different target groups regarding age, indicated by the arrow in the bottom of the visualisation

We identified eleven recommendations to strengthen alcohol health literacy and to reduce alcohol consumption in Germany that are summarized in Fig. 2. They are described below, separately for the three areas of action. For each recommendation, we cite key studies as example and highlight the aspects that need to be taken into account in order to achieve the intended effects.

### AREA 1: education and information measures

The recommendations concerning measures related to education and information are summarized in Table 1.

**Table 1:** Summary of recommendations related to education and information

#	Recommendation	Age of target population	Who will benefit the most from this measure?	Impact on antecendents, attributes or consequences of AHL (see [13])
1	Implement effective alcohol prevention programs in schools	Adolescents (12–17 years)	While everyone can participate, prevention programs are most effective for students abstaining or with low-risk alcohol use and less effective for those with established risky use	*Antecedents* (social and intervention level) *Attributes* (knowledge & understanding, skills, critical thinking) *Consequences* (reduced (risky) consumption, reduced under age consumption, improved health)
2	Provide easily-accessible information about alcohol use	In theory everyone but in practice mostly adolescents and young adults	Greater reach for people with higher education; mostly for persons abstaining or with low-risk alcohol use	*Antecedents* (system and social level) *Attributes* (knowledge & understanding; critical thinking)
3	Provide information material in simple language as well as in other languages than German, including but not limited to the internet	Total population	Persons with lower education and migration background; mostly for persons abstaining or with low-risk alcohol use	*Antecedents* (system and social level) *Attributes* (knowledge & understanding; critical thinking)
4	Health labels on containers of alcoholic beverages	No restrictions	No restrictions	*Antecedents* (system level) *Attributes* (knowledge & understanding, critical thinking) *Consequences* (reduced alcohol consumption)

#### #1 Implement effective alcohol prevention programs in schools

School-based alcohol prevention programs can be delivered by teachers or peers and may involve individualized interventions, role plays or internet content [26]. On review of the international literature, the effectiveness could not be established for many programs, but the “Unplugged” program has been singled out as good-practice for Europe, which focusses on knowledge and skills in a series of 12 sessions delivered over 1 year ([26], see also https://www.eudap.net/). Moreover, there are promising effects of web-based programs (although recent technological advances are not yet captured in the literature [27]). A good practice example from Germany is the program “Klar bleiben” (German for “stay clear”). In this intervention, students commit themselves to avoid engaging in heavy episodic drinking for a period of 9 weeks, in addition to discuss alcohol-related topics in four sessions (norms, motives, marketing, expectancies and consequences) [28].

For this recommendation, we refer to programs with established efficacy with regards to knowledge, skills, consumption and/or health outcomes. Such programs often contain modules that demonstrate how health risks depend on the amount of alcohol consumed or modules aiming to build skills for dosing alcohol as desired (e.g., understand how alcohol content and pouring size are related via the concept of standard drinks). Some of these programs also include modules to critically reflect on alcohol advertising (e.g. [28]), which are also called alcohol media literacy trainings [29]. Importantly, the programs need to be tailored to the pupils’ age in order to formulate appropriate aims (foster moderate drinking for older vs delaying onset of use for younger) and methods.

#### #2 Provide easily-accessible information about alcohol use

Access to accurate information on alcohol use and health implications is a prerequisite for developing AHL but the impact will depend on a number of factors. Public educational campaigns are a key measure here. While effects are generally difficult to establish, two separate campaigns have been related to increased awareness of alcohol as cancer risk factor and elevated support for alcohol control policies [30, 31]. In Germany, the widely known and ongoing campaign “Kenn dein Limit” (German for “Know your Limit”; [32]) informs people about the health risks of alcohol and calls for moderating one’s use mostly via public information materials, short educational videos, and a campaign website.

It should be noted that educational campaigns are very limited in the amount of information they can convey. Accordingly, they need to be diversified in their modalities including interactive formats (e.g., chatbots) to ensure a wide reach and higher engagement and should be accompanied by a resource pool (e.g., on the campaign website) that can be easily accessed by recipients for further information.

#### #3 Provide information material in simple language as well as in other languages than German, including but not limited to the internet

Currently, there is limited alcohol-related information (health risks, explanation of standard drinks) in simple language, or languages other than German, from official sources. In order to reach disadvantaged populations, e.g., people with low literacy, and populations with a first language other than German, such information should be tailor made to reflect their needs.

#### #4 Health labels on containers of alcoholic beverages

There are promising outcomes of visible and easy-to-understand warning labels with regards to increasing knowledge of health risks [33] as well as reducing alcohol sales [34]. The impact of health labels on knowledge of the alcohol-cancer link was also found to be linked to elevated support for alcohol control policies [35]. Furthermore, labelling can facilitate the understanding of standard drinks and improve skills for accurate pouring, but should be accompanied by additional educational initiatives to ensure that skills are adequately built (see recommendation #1; [36, 37]). Also, communication of health risks should be accompanied by messages that increase self-efficacy [38], and labels should be tested for acceptability among drinkers prior to being implemented to ensure efficacy (e.g. [39, 40]). Lastly, this recommendation is in line with Europe’s Beating Cancer Plan, through which the European Commission plans to adopt a proposal for health warnings on alcoholic beverages [41].

### AREA 2: Health care system measures

The recommendations concerning health care system measures are summarized in Table 2.

**Table 2:** Summary of recommendations related to the health care system

#	Recommendation	Age of target population	Who will benefit the most from this measure?	Impact on antecendents, attributes or consequences of AHL (see [13])
5	Alcohol screenings and subsequent feedback/ intervention in routine health checks for persons aged 35 years or older (financed by statutory health insurance)	Persons > 35	Persons with high-risk alcohol use	*Antecedents* (service level) *Attributes* (knowledge & understanding, skills, critical thinking, system competence) *Consequences* (reduced consumption, improved health)
6	Alcohol screenings for persons newly admitted to nursing homes/facilities	Mostly older adults	Persons with high-risk alcohol use	*Antecedents* (service level) *Attributes* (knowledge & understanding, skills, critical thinking, system competence) *Consequences* (reduced consumption, improved health)
7	Adequate financing/supply of resources for alcohol screenings and brief interventions in primary health care settings	Mostly adults	Persons with high-risk alcohol use	*Antecedents* (service level)
8	Training of primary health care professionals for implementing routine alcohol screenings and brief interventions	Mostly adults	Persons with high-risk alcohol use	*Antecedents* (service level)

#### #5 Alcohol screenings and subsequent feedback/ intervention in routine health checks for persons aged 35 years or older (financed by statutory health insurance)

Screening for high-risk alcohol use using short, standardized instruments like the three-item version of the Alcohol Use Disorder Identification Test is recommended by the German Guidelines on Screening, Diagnosis and Treatment of Alcohol Use Disorders (high-risk definition according to guideline: women  ≥ 12 g / men  ≥ 24 g pure alcohol intake per day [42]). While alcohol screening is rarely conducted in German primary health care settings, increased screening activities have the potential to lower population-level alcohol consumption [43]. There is already a routine health checkup for adults aged 35 and older [44] and alcohol should be added to this checkup.

While screening in itself may not foster AHL, it facilitates a conversation on health risks and possibly referral to specialist treatment if screened positive. Interventions subsequent to screening may be based on a continuum of alcohol health risk rather than a binary disease model, to increase problem recognition in patient populations [45]. For patients screened negatively, the treating physician would usually give positive feedback, which could function as reinforcement for low-risk alcohol consumption and further create a supportive environment for AHL.

#### #6 Alcohol screenings for persons newly admitted to nursing homes/facilities

As above, routine alcohol screening may serve AHL through various pathways. Routine alcohol screening and subsequent brief interventions are feasible in nursing homes and can be conducted by nurses with additional training. Among older adults, high-risk alcohol use is more often unrecognized [46], however, a small minority of older adults who drink heavily may require specialist care that may not be available in the nursing home. Here, sufficient capacities for supporting the patient in achieving abstinence or other treatment goals [47] are required and should be planned ahead, including medications for withdrawal treatment, as well as psychosocial support [42, 48].

#### #7 Adequate financing/supply of resources for alcohol screenings and brief interventions in primary health care settings

Routine screening for alcohol use and the delivery of subsequent brief interventions requires a substantial level of capacities in primary health care settings. Previous research has demonstrated that lack of financial incentives constitute a main barrier for alcohol screening and brief interventions [49] and withdrawal of adequate financing was related to a reduction of alcohol screenings in England [50]. Improved financing will not impact AHL directly but is a requirement for recommendation #5 and #6.

#### #8 Training of primary health care professionals for implementing routine alcohol screenings and brief interventions

In Germany but also most other countries, heavy drinking and alcohol use disorders are not commonly managed in primary health care setting [51, 52]. The importance for routine alcohol screening are often neglected by German primary healthcare professionals, while in other countries, stigma-related barriers appear to be more prevalent [53]. Given this, it is not surprising that the training of health care staff can improve alcohol management [54]. Training may not only increase the alcohol-related knowledge and skills of health care workers but also increase their self-efficacy to bring up this topic with patients. As part of training, protocols for management of acute cases, including acute effects of alcohol withdrawal, need to be developed.

As with recommendation #7, training health care staff is not expected to impact on AHL in the general population directly, but it is a prerequisite for recommendation #5 and #6.

### AREA 3: alcohol control policy measures

The recommendations concerning alcohol control policy measures are summarized in Table 3. Importantly, alcohol control policy measures may not directly target the core attributes of AHL which lie within the individual, but they can create a supportive environment facilitating AHL. For example, if students are taught that alcohol is harmful and they should not drink and drive but at the same time alcohol is being sold around the clock at gas stations, this could impair a coherent understanding of alcohol-attributable harms.

**Table 3:** Summary of recommendations related to alcohol control policy

#	Recommendation	Age of target population	Who will benefit the most from the recommended measure?	Impact on antecendents, attributes or consequences of AHL (see [13])
9	Reduction of the (spatial/temporal) availability of alcoholic beverages	No restrictions	No restrictions	*Antecedents* (system and social level) *Consequences* (reduced consumption; improved health; reduced underage consumption)
10	Raising alcohol taxes	No restrictions	Persons with current use, lower effect for persons with high-risk use	*Antecedents* (system and social level) *Consequences* (reduced consumption; reduced under age consumption; improved health)
11	Bans on alcohol advertising	Technically everyone but effects are mostly restricted to adolescents	No restrictions	*Antecedents* (system and social level) *Consequences* (reduced under age consumption)

#### #9 Reduction of the (spatial/temporal) availability of alcoholic beverages

Currently, there are no temporal or geographical restrictions to sell alcoholic beverages in most German jurisdictions. However, a natural experiment conducted in one region in south-west Germany showed that banning late-night off-premises alcohol sales is linked to improved health outcomes and reduced crime [21, 55]. Other (quasi-) experimental studies further show that alcohol consumption increases when the number of days on which alcoholic beverages can be sold is expanded [56]. In addition to temporal restrictions, there is solid evidence that a higher density of alcohol outlets is linked to higher crime rates [57].

#### #10 Raising alcohol taxes

Increasing alcohol taxes also does not directly target the core attributes of AHL. As with reduced availability, increasing retail prices by increasing taxes is expected to result in reduced consumption and improved health [58, 59]. Currently, alcohol taxes in Germany are among the lowest in Europe [60], resulting in alcoholic beverages to be more affordable in Germany than in most other countries [61]. To justify a hike in retail prices driven by tax increases, policymakers would likely refer to the health risks carried by alcoholic beverages—a similar reasoning as employed by the European Commission [41]. A public debate involving alcohol-related health risks may facilitate a supportive environment for developing AHL.

#### #11 Bans on alcohol advertising

Banning advertising for alcoholic beverages does not directly target the core attributes of AHL. While the evidence is not clear that a ban is directly impacting on alcohol use among adults [62], it can be reasonably assumed that alcohol marketing is facilitating underage alcohol use, e.g. by creating positive social norms regarding alcohol use [63]. In addition to positive effects on consumption, a marketing ban would reduce the need for critical thinking (e.g., deconstructing the marketing intent) resulting in less interference in individuals decision-making process (i.e., more direct transfer of knowledge to behavior).

### Expert rating of the recommendations

**Fig. 3:**
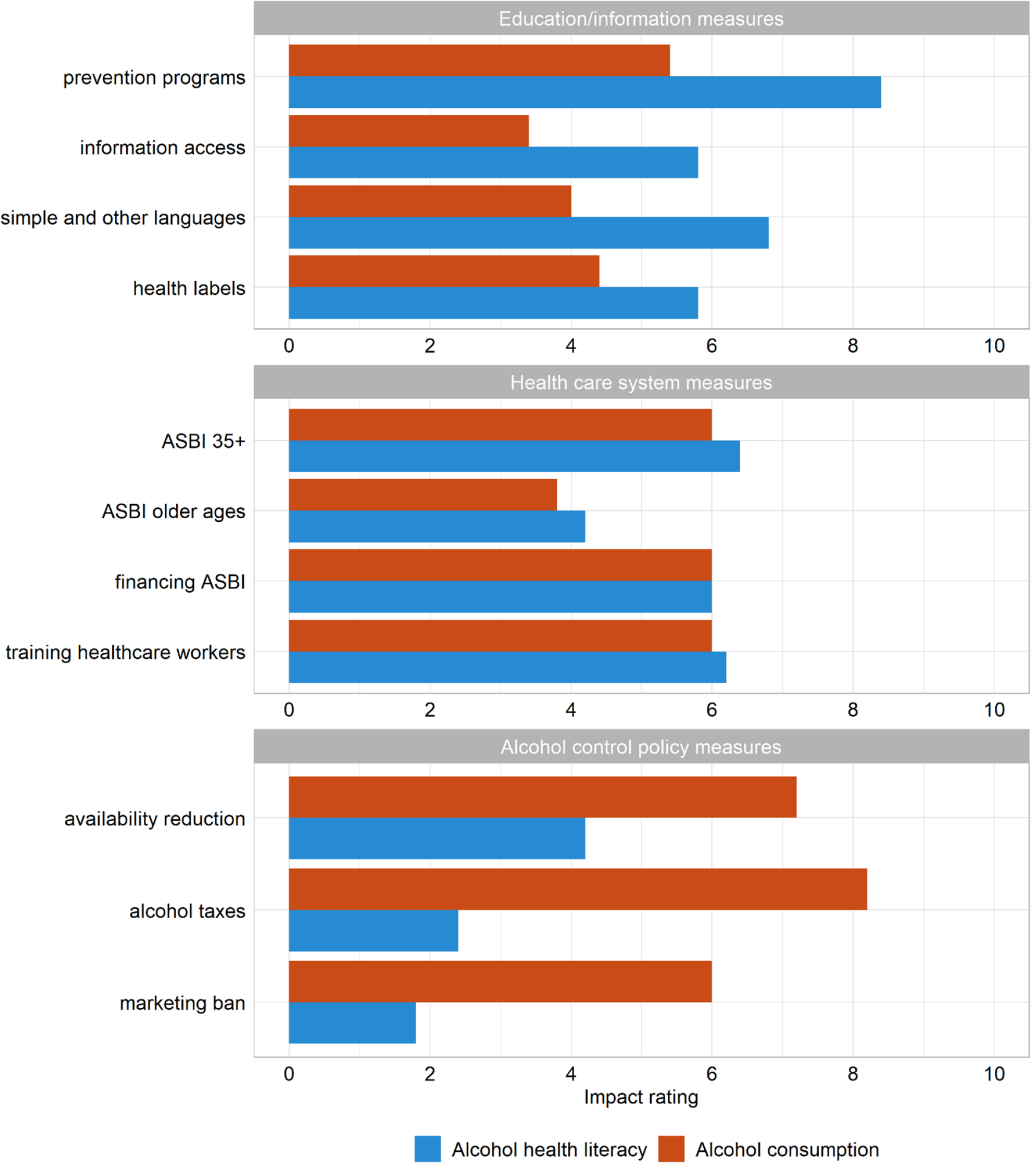
Impact of recommended measures on alcohol health literacy (blue bars) and alcohol consumption (red bars), as rated by five experts, by area of action; *ASBI* Alcohol Screening and Brief Intervention

Each expert rated each of the eleven recommendations with regards to the likely impact on AHL and alcohol consumption on a scale of 1–10. For both AHL (ICC = 0.88; 95% confidence interval: 0.76–0.96) and alcohol consumption (ICC = 0.85; 95% confidence interval: 0.70–0.94), the ratings showed good agreements. The ratings are summarized in Fig. 3.

Overall, educational and health care system measures were rated to be most impactful for strengthening AHL. Of all recommendations, the experts highlighted the impact of effective prevention programs in schools (mean: 8.4) as well as the provision of information in simple and other languages than German (mean: 6.8). The three alcohol control policies were rated as least important for strengthening AHL (means between 1.8 and 4.2).

For reducing alcohol consumption, health care system and alcohol control policy measures were prioritized. The most impactful measures were to raise taxes (mean: 8.2) and to reduce the availability of alcoholic beverages (mean: 7.2). A marketing ban and the implementation of alcohol screenings and subsequent interventions in health check-ups for persons aged 35 or older were perceived as equally impactful (mean: 6.0).

## Discussion

In this study, we derived 11 recommendations to increase AHL and to decrease alcohol consumption in the German population. Notably, education-based measures, such as (school-based) prevention programs and health labels were judged to be more effective for raising individual competencies regarding alcohol consumption and harm, but less so for impacting on alcohol consumption itself. Conversely, the experts prioritised alcohol control policy measures, with alcohol taxes believed to be the most effective tool to curb consumption, while they are less likely to impact directly on AHL. While these recommendations were developed for Germany, they are likely to be applicable to other countries with cultural and economic similarities, such as most Central and Western European countries.

### Interpretation of the findings

The ratings from the experts suggest that strengthening AHL and curbing alcohol consumption may require two different pathways. While a more restrictive environment (marketing ban, decreased availability, increased taxes) would be expected to reduce alcohol consumption in some populations, it might not necessarily improve AHL, e.g., understanding of health risks or the skills to pour the desired amount of alcohol. Conversely, effective prevention programs and health labels on alcoholic beverage containers would be expected to enhance AHL but this may not necessarily be translated into alcohol use behavior.

This pattern could be interpreted as AHL being an entity that is separate from consumption. It appears that the literature supports this assessment: programs aiming to enhance AHL usually focus on psychological concepts, like awareness or skills (e.g., [29, 30]), while the evaluation of alcohol control policies are usually evaluated using sales figures or health outcomes (e.g., [55, 64]). This apparent distinction could be explained by the intervention aims which are determined by stakeholder interest. Arguably, creators of educational programs are sufficiently humble to not expect that their program will have a noticeable short-term impact on consumption, or they are genuinely more interested in psychological concepts that explain behaviour. Conversely, policymakers may not be interested to know whether risk awareness among alcohol users has increased following a price hike. Yet, if the (long-term) effects of educational programs on consumption are not measured and the role of AHL for the success of stricter control policies is ignored, these two areas of action will remain distinct limiting their potential effectiveness.

While we do not provide empirical evidence that these two areas of research need to be combined but the perceived discreteness of educational programs vs. control policies is contrasting the AHL conceptualization. According to the concept proposed by Okan and colleagues [13], the system level is an antecedent for developing AHL attributes. In other words, an environment conveying that alcohol is not an ordinary commodity but comes with certain social and health risks, should facilitate the learning of adequate cognitions and skills, such as risk awareness and pouring skills. Moreover, greater risk awareness can also pave the way for implementing stricter control policies.

Contrasting these theoretical assumptions, we have very limited *empirical* knowledge of how the alcohol control policies interacts with education-based measures. It could be conceived that raising alcohol taxes at the same time as launching a large-scale educational campaign (e.g., on risky drinking thresholds) may foster the understanding of alcohol-related health risks and thereby produce stronger effects regarding alcohol consumption than either of the two measures on their own. Based on these considerations, it appears reasonable for researchers to explore opportunities for overcoming the discreetness of these two areas of action in order to maximize the intendend effects.

While we have identified several recommendations that appear feasible for improving AHL and reducing consumption for various parts in the population, there is also some caution warranted that becomes apparent when drawing parallels to achievements regarding tobacco use. Unlike alcohol use, smoking has become considerably less prevalent in Germany in the past decades [65]. This progress likely is the result of a combination of several measures including control policies (e.g., price hikes and restrictions in places allowed to smoke) as well as educational measures (e.g., health warning labels [65]). While this strategy was overall successful, smoking behavior has shifted from populations with higher to populations with lower socioeconomic status [65], resulting in a major cause for health disparities [66]. In fact, it has been argued that strong tobacco control policies may result in stigmatizing those who keep smoking, which could introduce a barrier to healthcare access [67].

Learning from these experiences, we should avoid repeating these errors. On one hand, the tobacco experiences suggests that control policies alone may be more effective among people with higher socioeconomic status and can lead to segregation and thus stigmatization of users. On the other hand, development of health literacy is strongly dependent on resources such as educational and financial background [22]. For example, a major German educational campaign regarding alcohol risks is less commonly remembered by youth with migration background and among those not visiting higher educational institutions [68]. Thus, focussing on strengthening AHL alone may also result in widening the disparities with regards to alcohol use and consequences.

Whilst, unlike tobacco use, alcohol use in everyday life is currently well accepted and prevalent in all age, sex, and educational groups [69], alcohol use disorders are among the most stigmatized conditions, on par with schizophrenia [70]; it is assumed that this is one of the causes for the very low treatment rates for this condition [71]. The stigmatization of people with alcohol use disorders prevents those affected and health care providers engaging in honest and unprejudiced conversations on alcohol use.

Learning from the experience in smoking cessation, we propose that any measure aiming to tackling the societal alcohol burden should result in equitable reductions across socioeconomic groups without stigmatizing people who use alcohol or who have developed alcohol use disorders. Importantly, implementing the measures proposed should be monitored in this regard.

### Limitations

The recommendations presented in this study are derived from a non-systematic and undocumented search of the literature. While adjusted and extended based on the experts’ input, we cannot rule out a selection bias. Also, it is possible that the choice of experts involved in this study may have influenced the mean ratings. However, there was overall good agreement between the experts recruited from very different backgrounds, minimizing the risk that another group of experts would have come to a substantially different set of ratings.

## Conclusions

To achieve a sustainable improvement of AHL and reduction of alcohol consumption, a comprehensive alcohol strategy is required. Using Germany as an example, we have identified 11 recommendations likely to enhance AHL and to reduce alcohol consumption. Both aims—enhancing AHL and reducing consumption—should be considered complementary and not separately.

## Data Availability

The reponses of the experts collected and analysed during the current study are available from the corresponding author on request.
